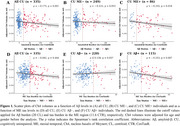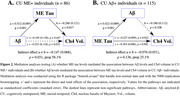# Early Tau and Amyloid Co‐Pathology Linked to Basal Forebrain Atrophy in Cognitively Unimpaired Older Individuals

**DOI:** 10.1002/alz70862_110840

**Published:** 2025-12-23

**Authors:** Ying Xia, Matthew Dean, Vincent Dore, Natasha Krishnadas, Pierrick Bourgeat, Paul Maruff, Colin L Masters, Victor L. Villemagne, Jurgen Fripp, Christopher C. Rowe, Elizabeth J Coulson

**Affiliations:** ^1^ CSIRO Health and Biosecurity, Australian E‐Health Research Centre, Brisbane, QLD Australia; ^2^ School of Biomedical Sciences, The University of Queensland, Brisbane, QLD Australia; ^3^ CSIRO Health and Biosecurity, Australian E‐Health Research Centre, Parkville, VIC Australia; ^4^ Austin Health, Heidelberg, VIC Australia; ^5^ The Florey Institute of Neuroscience and Mental Health, Melbourne, VIC Australia; ^6^ Cogstate Ltd., Melbourne, VIC Australia; ^7^ The Florey Institute of Neuroscience and Mental Health, The University of Melbourne, Parkville, Melbourne, VIC Australia; ^8^ University of Pittsburgh School of Medicine, Pittsburgh, PA USA; ^9^ Queensland Brain Institute, The University of Queensland, Brisbane, QLD Australia

## Abstract

**Background:**

The cholinergic basal forebrain (BF) system, particularly the nucleus basalis of Meynert (Ch4), is selectively vulnerable to early pathological changes in Alzheimer’s disease (AD). However, the onset and mechanisms of Ch4 atrophy in relation to AD pathologies are not well understood. This study investigated the relationships between Ch4 volume and amyloid‐β (Aβ) and tau pathologies in cognitively unimpaired (CU) older individuals, where early‐stage pathology enables a better characterisation of Ch4 degeneration development.

**Method:**

The cross‐sectional study included 335 CU individuals (74.1 ± 6.3 years old, 56.7% female) from the Australian Imaging, Biomarkers and Lifestyle (AIBL) study who underwent PET imaging for Aβ (18F‐NAV4694) and tau (18F‐MK6240) and MRI during the same visit. Tau burden was quantified in the mesial‐temporal (ME) region using CapAIBL and expressed in CenTauR (CTR). Thresholds for Aβ positivity (Aβ+) and TME tau positivity (ME+) were defined as 20 Centiloids and 11.6 CTR (1.5 standard deviations above CU Aβ− individuals), respectively. MRI‐derived Ch4 volumetric measures were calculated and adjusted for age and gender using the sample of CU Aβ− ME− individuals. Associations between Aβ burden, ME tau levels, and Ch4 volumes were evaluated.

**Result:**

In CU individuals, Ch4 volume was associated with Aβ levels, but not with ME tau burden (Figure 1A, 1D). When individuals were stratified by ME tau status, Ch4 volume showed no significant association with Aβ levels in CU ME− individuals. However, in CU ME+ individuals, Ch4 volume was associated with Aβ levels (Figure 1C), with ME tau levels mediating 58.1% of the total effect (β[SE] = −0.147[0.068], *p* = 0.031; Figure 2A). Similarly, when individuals were stratified by Aβ status, Ch4 volume correlated with ME tau levels only in the presence of Aβ pathology (Figure 1F), while the Aβ burden did not significantly contribute to this indirect effect (β[SE] = −0.076[0.051], *p* = 0.136; Figure 2B).

**Conclusion:**

The co‐occurrence of amyloid and tau pathologies was associated with reduced Ch4 volume. Without detectable amyloid pathology, Ch4 is not vulnerable to the mesial‐temporal tau pathology. However, in the presence of amyloid pathology, tau pathology may have a more direct role in Ch4 degeneration.